# The *Troika* Host–Pathogen–Extrinsic Factors in Tuberculosis: Modulating Inflammation and Clinical Outcomes

**DOI:** 10.3389/fimmu.2017.01948

**Published:** 2018-01-09

**Authors:** Helder Novais Bastos, Nuno S. Osório, Sebastien Gagneux, Iñaki Comas, Margarida Saraiva

**Affiliations:** ^1^Department of Pneumology, Centro Hospitalar do São João, Porto, Portugal; ^2^Life and Health Sciences Research Institute (ICVS), School of Medicine, University of Minho, Braga, Portugal; ^3^ICVS/3B’s—PT Government Associate Laboratory, Braga, Portugal; ^4^Department of Medical Parasitology and Infection Biology, Swiss Tropical and Public Health Institute, Basel, Switzerland; ^5^University of Basel, Basel, Switzerland; ^6^Institute of Biomedicine of Valencia (IBV-CSIC), Valencia, Spain; ^7^CIBER of Epidemiology and Public Health (CIBERESP), Madrid, Spain; ^8^i3S—Instituto de Investigação e Inovação em Saúde, University of Porto, Porto, Portugal; ^9^Instituto de Biologia Molecular e Celular (IBMC), University of Porto, Porto, Portugal

**Keywords:** tuberculosis, genotypic diversity, immune phenotypes, severity of disease, inflammation, microenvironments

## Abstract

The already enormous burden caused by tuberculosis (TB) will be further aggravated by the association of this disease with modern epidemics, as human immunodeficiency virus and diabetes. Furthermore, the increasingly aging population and the wider use of suppressive immune therapies hold the potential to enhance the incidence of TB. New preventive and therapeutic strategies based on recent advances on our understanding of TB are thus needed. In particular, understanding the intricate network of events modulating inflammation in TB will help to build more effective vaccines and host-directed therapies to stop TB. This review integrates the impact of host, pathogen, and extrinsic factors on inflammation and the almost scientifically unexplored complexity emerging from the interactions between these three factors. We highlight the exciting data showing a contribution of this *troika* for the clinical outcome of TB and the need of incorporating it when developing novel strategies to rewire the immune response in TB.

## Introduction

According to current estimates, tuberculosis (TB) accounted for approximately 1.7 million deaths in 2016 and affected one-quarter of the world’s population in its latent form (1). TB is a heterogeneous disease, characterized by a continuous spectrum of infection, for which molecular and clinical biomarkers of progression are just starting to be unveiled. Several stages of latent TB infection (LTBI) exist and include subclinical forms of TB with an increased likelihood of progressing to active disease (2, 3). The clinical manifestation of the active disease is highly variable, with mild or extensive pulmonary involvement, extrapulmonary, or disseminated forms of TB. Many known TB precipitating factors, which either increase the susceptibility to TB or the risk of transition from LTBI to active TB, are connected with immune imbalances (4, 5). However, the molecular mechanisms governing the transitions along the TB spectrum remain unknown.

**Figure 1:**
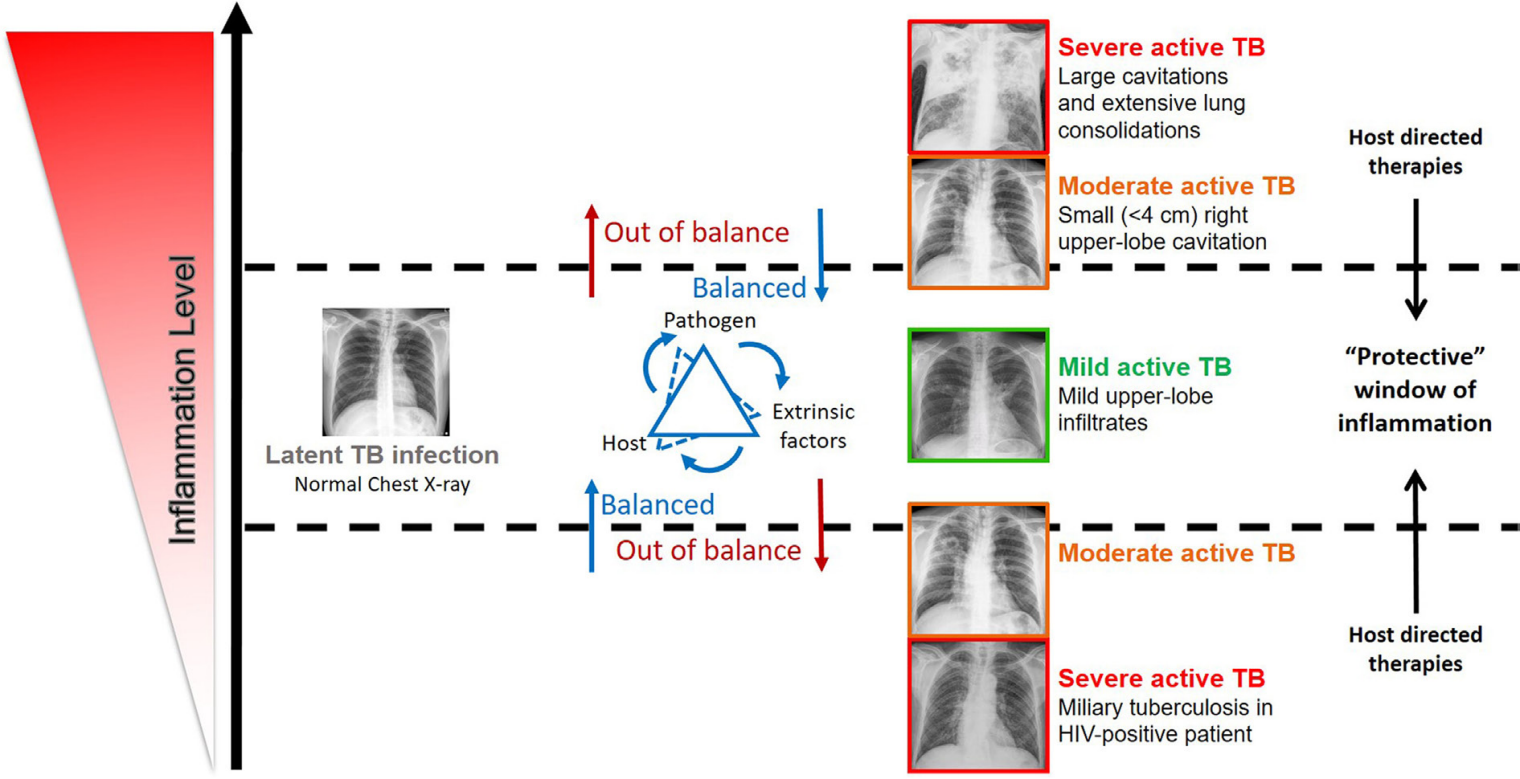
Impact of the inflammation level in the disease outcome in individuals infected by *Mycobacterium tuberculosis* complex bacteria. The spectrum of tuberculosis (TB) disease is strongly linked with the host immune status. The inflammation level results from the interaction of host, pathogen, and extrinsic factors. Very low and high inflammation levels often associated with severe active TB, while balanced immune responses associated with mild active TB, latent TB, and possibly TB clearance. Evidence supports that host-directed therapies (see Table 1) have the potential to successfully modulate inflammation and ameliorate disease outcome, by ensuring a protective immune response.

The immune condition of infected hosts is shaped by genetics, extrinsic factors altering the local microenvironment, and the heterogeneity of the infecting bacteria. This *troika* determines the threshold of the immune response generated during infection and possibly the disease outcome (Figure 1). Modulating these thresholds and uncovering the links between host, pathogen, and microenvironments should allow for the discovery of solid correlates of protection, molecular markers for disease prognosis, and the development of safe and effective host-directed therapies (HDTs) to TB. This review covers our current understanding of the impact of these elements on TB, their interactions, and how they may be further explored as a platform for developing new products and strategies against TB.

## Host Genetics: Role in Immunomodulation

The association of single-gene inborn errors related to interferon (IFN)-γ immunity with severe childhood TB provides the clearest genetic basis for TB susceptibility (6, 7). These conditions, globally named Mendelian susceptibility to mycobacterial disease (MSMD) encompass a series of germline mutations in seven autosomal (*IFNGR1, IFNGR2, IL12B, IL12RB1, STAT1, IRF8*, and *ISG15*) and two X-linked (*NEMO* and *CYBB*) genes (6, 7). These defects are functionally and physiologically related, as they all result in an impairment of the CD4 T cell-mediated immunity and have provided decisive evidence on the critical protective role of the interleukin (IL)-12/IL-23/IFN-γ loop in TB (6, 7). This role was further confirmed in the context of secondary immunodeficiencies, such as in human immunodeficiency virus (HIV) infection, as discussed below.

Genetic association studies with adult patients showed more limited success than MSMD, and strikingly no consistent association of variants of genes from the IL-12/IFN-γ axis with TB susceptibility in adulthood was found. Instead, candidate-based studies found a number of genetic variants associated with TB susceptibility in humans (7–9). However, results remain inconsistent and have not been validated in different populations nor in genome-wide association studies (GWAS) (10–12). This most likely reflects the association of adult pulmonary TB with complex genetic traits, where the role of genetic–extrinsic factors and gene–gene interactions (epistasis) dominate over single polymorphisms on their own (13, 14). The identification of genetic risk factors is also likely masked by the experimental design, where important contributors, such as extrinsic factors, or pathogen variability are largely neglected. Furthermore, the full spectrum of TB has been mostly ignored in the group definition, with all phenotypes being analyzed together in two main study groups: active TB versus healthy controls/LTBI. Finally, combining and integrating genetic association studies with the investigation of the human epigenome will certainly lead to critical insights into the genetic basis of infection and clinical TB.

## The Modulation of the Immune Response by Extrinsic Factors

**Figure 2:**
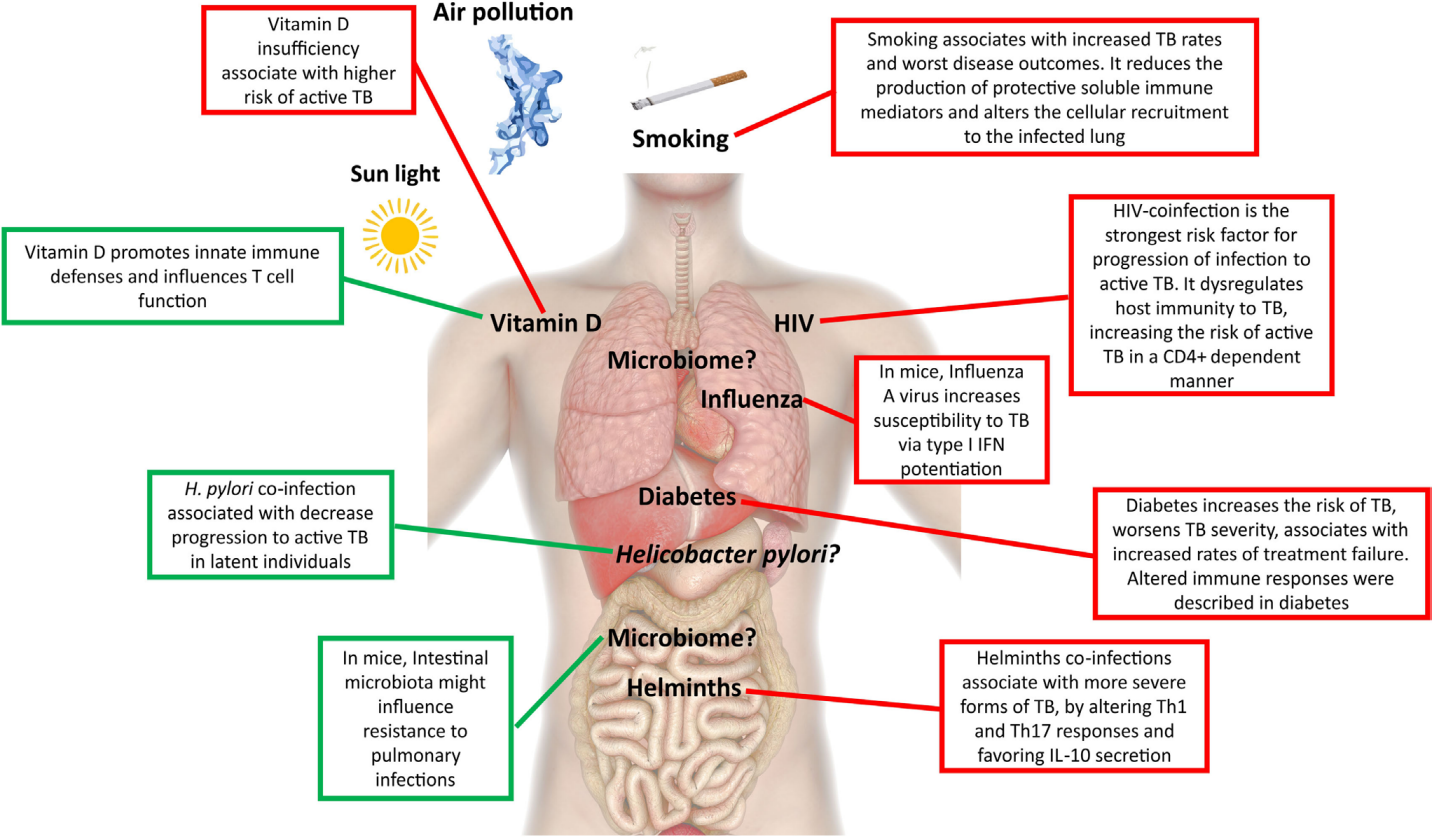
Extrinsic factors associated with active tuberculosis (TB). This figure depicts extrinsic factors associated with protection to active TB (green) or susceptibility/increased risk (red) for active TB development.

The lack of clear association of human genotypes with TB susceptibility and the fact that progression from LTBI to pulmonary TB in adults usually reflects an impairment of host resistance due to non-genetic factors, highlighting the relevance of extrinsic factors in shaping the host immunity with an impact on TB outcome (Figure 2).

### Coinfections

A major driver of the current TB epidemics has been the HIV syndemic, which dramatically decreases the host protective responses to TB in a CD4 T cell count-dependent manner (15), leading to an acceleration of both diseases. Because the risk of developing TB is largely increased in HIV-infected individuals even before CD4 T cell counts decrease (16), other factors must also play a role. Indeed, a detrimental alternative activation of macrophages, accompanied by less nitric oxide (NO) synthase expression and poorly formed granulomas was described in HIV–TB, which in turn downregulated the *Mycobacterium tuberculosis* DosR regulon (17), a set of genes known to be induced during anaerobic dormancy (18). Therefore, changes in the host immunity resulting from HIV coinfection remodel the bacterial physiology, further rewiring the host tissue microenvironment. Consequently, the pathogenesis of TB is different in HIV-coinfected individuals, resulting in the lack of complete cavitation and in a higher incidence of disseminated disease (19, 20). Other less studied coinfections with impact on TB include helminths, influenza, and *Helicobacter pylori*. In TB patients coinfected with helminths, a more advanced form of disease was reported (21), possibly related with decreased T helper (Th) 1 and Th17 cell responses and increased secretion of IL-10 (22, 23). In the case of influenza, the increased susceptibility to *M. tuberculosis* infection is likely mediated by type I IFN signaling (24). Contrastingly, infection with the ubiquitous bacteria *H. pylori* may help to avoid progression to active TB in latent individuals, due to enhanced IFN-γ and other Th1-like cytokine responses generated in response to *H. pylori* and that restrain *M. tuberculosis* (25). Another example of coinfection cross talk in TB comes from the mouse model, in which prior *Helicobacter hepaticus* colonization impaired the immune control of *M. tuberculosis* (26). It is interesting that of these three infections, only influenza is also a lung disease, thus indicating that distant events shape the lung microenvironment.

### The Host Microbiome

Different mouse models of infection depleted of commensal gut microbiota after antibiotic treatment showed an increased risk of colonization by respiratory pathogens, such as *Streptococcus pneumoniae* (27), *Staphylococcus aureus* (28), and *Klebsiella pneumoniae* (29). Alterations in the gut microbiota also alter the susceptibility to TB (30), and an increase in the lung bacterial burden early post *M. tuberculosis* infection was reported in germ-free mice (31). Limited available data suggest that the gut microbiota of mice infected with *M. tuberculosis* is drastically reduced after initial infection (32). Gut diversity in *M. tuberculosis*-infected mice is recovered about the time that the adaptive immune system is onset, although significant differences in taxa composition remain in pre- and postinfection samples (32). In addition, experiment removal of the gut microbiota with antibiotic treatment leads to higher susceptibility to *M. tuberculosis* infection in mice (30). How the intestinal microbiota distally affects pulmonary immunity, and eventually the course of infection and disease, and how the gut–lung axis may impact TB await further research.

We are also still far from understanding the composition and the impact of changes in the oral–nasal cavity and lung microbiota on TB. *M. tuberculosis* establishes infection in the lower respiratory tract, and as such has to initially evade microbiota-activated macrophages of the upper respiratory tract (33). It remains to be seen if the presence or absence of certain microbial species in the upper respiratory airway generates a more permissive environment for the establishment of TB infection. If this is the case, restoring key players of the microbiota may help to fight invasion or even improve immunity, therapeutic possibilities not yet explored in TB infection.

### Non-Communicable Comorbidities

Among non-communicable comorbidities, the presence of diabetes remains the major risk factor for TB. Owing to the dimension of the diabetes epidemics, its foreseen impact on the global numbers of TB cases is alarming. Diabetic patients have a three times higher risk of developing TB than healthy individuals (34). Diabetes also worsens disease severity (35), is a risk factor for death in TB patients (36, 37), and is associated with increased failure of standard TB treatment (35, 38, 39). Previous studies suggest that the interaction of *M. tuberculosis* with macrophages and dendritic cells (DCs) is impaired in the context of diabetes, leading to an initial hypo-inflammatory state (40–43). Once the infection is established, there is evidence for increased inflammation in TB/diabetes patients, as an augmented level of pro-inflammatory cytokines in the peripheral blood is measured, likely due to hyperactive T cell responses (41–43). It is possible that many of the alterations seen and their impact on TB are actually interconnected to changes in the composition of the human gut microbiota imposed by diabetes (44).

Subjects with chronic obstructive pulmonary disease (COPD) present greater risks for developing active TB (45, 46) and TB death as compared with TB patients without this comorbidity (36, 45). Altered immune responses likely underlie the mechanisms linking COPD and TB. COPD is characterized by a disruption of innate defense mechanisms in the airways, including decreased mucociliary clearance and impaired macrophage phagocytosis (47, 48). Furthermore, an accumulation of lung regulatory T cells and the increase of circulating IL-10 and TGF-β (49) were described in COPD patients. Thus, COPD limits the effector function of T cells in response to pathogens, which may explain the increased susceptibility to lower respiratory tract bacterial infections, including *M. tuberculosis*.

### Environmental Factors

Smoking exposure is an independent risk factor for *M. tuberculosis* infection, progression to active disease, and for poor treatment outcomes (50–52). The underlying immunological mechanisms are just starting to be unveiled and include reduced production of tumor necrosis factor (TNF), IL-1β, and IFN-γ by *in vitro* infected alveolar macrophages (53), decreased number of DCs (54, 55), and compromised recruitment of IFN-γ-producing CD4 T cells to the lung, thus weakening the formation of granuloma (56). More recently, alveolar macrophages from smokers were found to exhibit lysosomal accumulations of tobacco smoke particulates, which impaired their migration toward *M. tuberculosis*-infected cells (57).

Malnutrition has also been associated with an increased risk of active TB, although it remains unclear whether the nutritional status is a cause or a consequence of the disease. A strong link between TB, malnutrition, and immune dysregulation is in place (58). This association is even worse in the framework of HIV infection (59). Nutritional status not only affects the function of several immune cells, including T cells, but additionally impacts the pharmacodynamics of the drugs (60). Less clear is the role of anemia, a common symptom, and prognosis marker of TB (36), particularly linked to iron deprivation, in facilitating or exacerbating TB disease. An emerging role is being recognized for hepcidin, a protein that regulates the homeostasis and cell type distribution of iron in the body and that plays a role on innate immune responses to mycobacterial infection (61, 62). In agreement, in epidemiological studies hepcidin levels have been positively correlated with increased risk of mortality in TB–HIV coinfection usually associated with more death-threatening manifestation of the disease, as extrapulmonary and miliary TB (63). Another nutrient, vitamin D, has been the focus of renewed attention by researchers. Historically, both vitamin D and exposure to sunlight, which endogenously promotes the conversion in the skin of 7-dehydrocholesterol into pre-vitamin D_3_, were used in the treatment of TB (64). Insufficiency of this molecule has been linked to higher risk of active TB (65, 66) and increased propensity for extrapulmonary involvement (67). The immunomodulatory role of vitamin D is well established leading to several changes in immune responses, including the induction of cathelicidin antimicrobial peptide, beta-defensin, and the promotion of authophagy and/or bacterial killing (68, 69). However, it is important to mention that despite several reports on the protective role for autophagy during *M. tuberculosis* infection (70), a recent study based on a genetic approach targeting multiple autophagy-related genes concluded that the cellular autophagic capacity did not correlate with the outcome of *M. tuberculosis* infection (71). Therefore, it is possible that the impact of autophagy during *M. tuberculosis* infection results from the use of *in vitro* models, thus calling for further *in vivo* studies when correlating protective mediators with induction of autophagy. Vitamin D levels have been shown to impact adaptive immune responses by influencing Th cell function and by promoting Tregs (72). It thus seems that vitamin D promotes the macrophage effector function, while at the same time keeping the immune response at check through its action on T cells. Several clinical trials have shown that supplementation of drug regimens with vitamin D does not improve TB outcomes in the general population (73), but it has been shown to accelerate sputum conversion in patients with the *tt* genotype of the *Taql* vitamin D receptor polymorphism (74). It remains to be seen if vitamin D can play a role in preventing infection or progression to active TB. It is possible that through the modulation of vitamin D, variations in sunlight may underlie some of the differences in TB incidence rate across the globe (65). This not only includes natural seasonal variations but also artificial variation due to human-associated activities, such as pollution.

## *M. tuberculosis* Diversity and Immunomodulation: Paradigm Changing Evidence

### How Variable Is *M. tuberculosis*?

**Figure 3:**
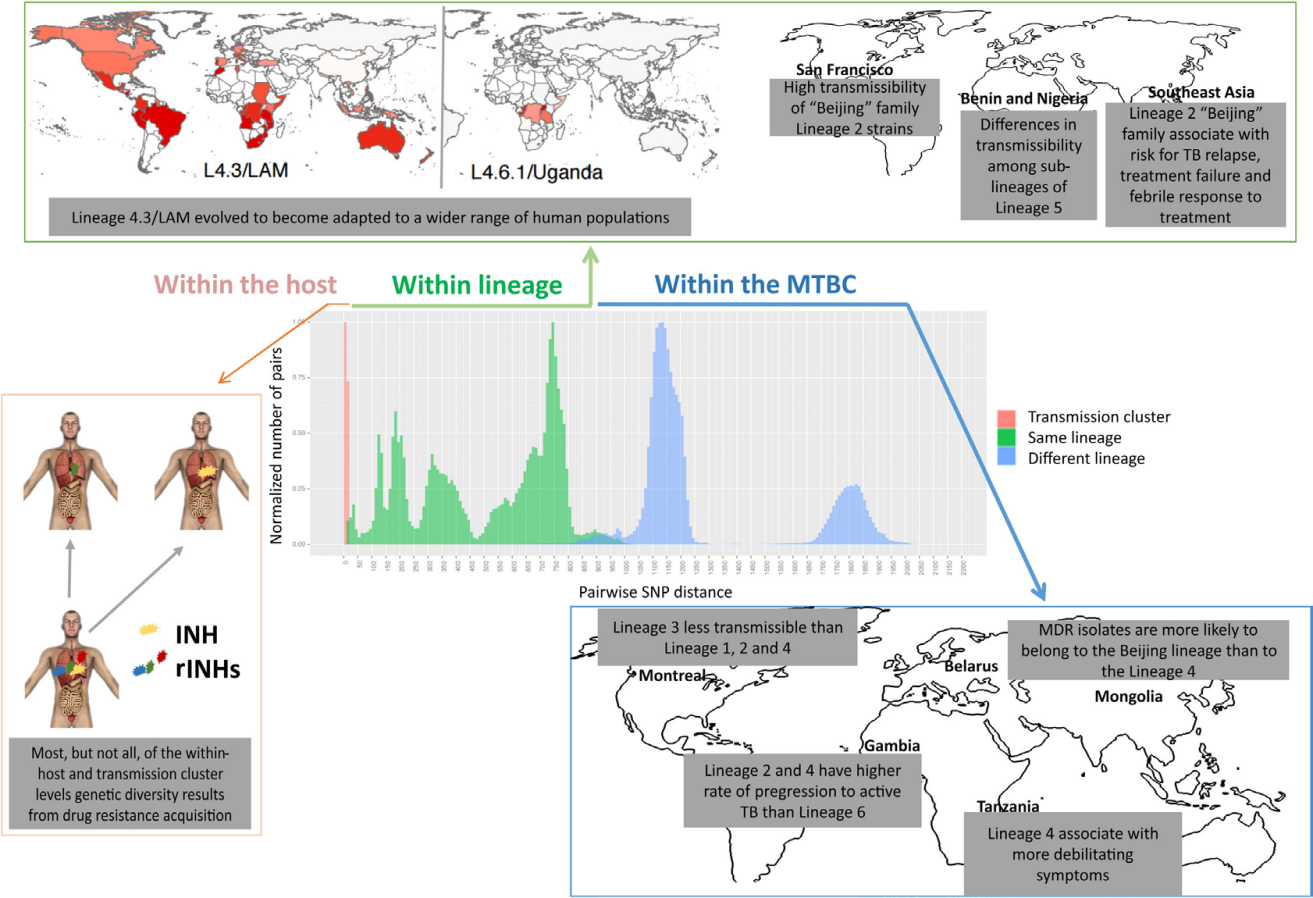
Levels of genetic diversity across the *Mycobacterium tuberculosis* complex (MTBC) and its epidemiological and clinical impact. There are different levels of diversity across the MTBC. Within a host or transmission chain, *M. tuberculosis* isolates typically differ in less than 25 single-nucleotide polymorphisms (SNPs). Diversity increases when comparing isolates within the same lineage (around 25–1,000 SNPs) or within different lineages of the MTBC (around 1,000–2,000 SNPs). This diversity impacts host/pathogen interactions, particularly the intensity and quality of the immune response and the clinical outcome, at the levels of drug acquisition, adaptation to different populations, transmissibility, or disease manifestation.

Tuberculosis is caused by a group of phylogenetically closely related bacteria, collectively known as the *M. tuberculosis* complex (MTBC), now known to encompass seven main phylogenetic lineages of human-adapted bacteria (75). Within this complex, *M. tuberculosis* and *Mycobacterium africanum* (in West Africa) are responsible for the large majority of human cases of TB (76). The genetic diversity within the MTBC is higher than originally expected and can be observed at different evolutionary and geographical scales (Figure 3). Most of the diversity observed is likely due to genetic drift, i.e., stochastic variation of diversity due to limited population sizes, or to neutral variation with no impact on the fitness of the bacteria (77). Diversification of the initial infecting bacteria in subpopulations within a single patient has also been reported (78), part of it is likely due to antibiotic selection pressures (79, 80). Although still unclear, additional diversity maybe selected independently of antibiotic pressure (80). It is possible that some of the diversity within the host reflects the heterogeneous immune responses associated with different lung lesions (81). The complexity of the lung microstructure is now acknowledged, with the immune response being spatially separated even in single granulomas (82).

### What Evolutionary Forces Shape MTBC Diversity?

In the long-term evolution of the pathogen, the main driving forces shaping MTBC diversity were a balance between genetic drift, during the global expansion of the bacteria and host-to-host transmission, and positive and purifying selection (77, 83). A long period of parallel evolution of the pathogen with different populations of modern humans following the out-of-Africa migrations (84) resulted in different bacterial lineages to prevail in different geographical regions of the world. Echoes of this type of interaction are still observed in cosmopolitan settings, where the phylogeny of the infecting MTBC isolates correlates with the patient’s ethnic origin (85, 86). A recent study showed that even within one specific MTBC lineage, i.e., Lineage 4 (or Euro-American), a phylogeographical structure can be found, with some sublineages being geographically restricted and others occurring globally (87).

Whether the immune system is also a driving force for *M. tuberculosis* diversity remains unclear. On one hand, extraordinary insights can be gained, as shown by the identification of single nucleotide polymorphisms (SNPs) in the two-component regulation system PhoP/PhoR impacting Lineage 6 and *Mycobacterium bovis* strains (88). On the other hand, while most pathogens have evolved to evade host immunity by antigenic variation, *M. tuberculosis* seems to apply a different strategy, thus questioning the role of T cells in driving *M. tuberculosis* evolution. Genome sequencing of *M. tuberculosis* showed that the known human T cell epitopes are evolutionarily hyperconserved, with the large majority of individual epitopes analyzed showing no amino acid change at all (89). It is interesting to note that epitopes appear to be significantly more conserved than the mean of the genome, and this result is robust to the number of strains analyzed (89, 90). Furthermore, these hyperconserved epitopes fall on genes that are not biased toward particular functional categories, but in which the major common theme is that they encode peptides that are recognized by the immune system (90). This hypothesis still awaits functional and experimental confirmation, but based on *in silico* analyses it is tempting to speculate that, despite the general conservation of the *M. tuberculosis* genome as compared with other bacteria, T cell responses may drive selective forces toward the hyperconservation of epitopes. The relevance of host T cell responses for the natural history of *M. tuberculosis* infection comes from findings in HIV+ patients, showing that less frequent cavitation, in a CD4 T cell-dependent frequency, associates with lower transmissibility (as inferred by looking at the number of household contacts infected by an HIV+ patient) (15). Therefore, it is conceivable that the T cell response that locally tries to eliminate the bacilli, which results in cavitation and thus in transmission. Ensuring conservation of T cell responses could thus be a mechanism explored by *M. tuberculosis* to ensure transmission. Nonetheless, outlier epitopes to the general rule of hyperconservation have been described both in previously known and newly identified antigenic regions (89, 91). Moreover, amino acid substitutions in these variable epitopes were shown to impact the host response with some patients responding only to the wild-type epitope variant and others only to the mutated forms (89). This is important, as the alteration of a single amino acid in an epitope can impact its affinity to a specific HLA molecule, thus influencing the T cell synapse and modulating the level and type of immune response elicited (92, 93). These variable epitopes could be potentially exploited as vaccine components to increase the protection provided by the existing TB vaccine.

### Impact of Bacteria Genotypes on Immune Responses and Clinical Outcomes

*Mycobacterium tuberculosis* complex diversity impacts the host immune response, as certain clinical isolates are more potent than others in inducing the secretion of immune mediators by infected monocytes (94–99) and in experimental infections (100–104). The impact of bacterial diversity is not only reflected at the level of soluble immune mediators but also in the ability of virulent mycobacteria to inhibit apoptosis, while triggering necrosis of host macrophages to promote an innate delay in the initiation of adaptive immunity (105). Clearly, unbalancing the immune response is a strategy used by *M. tuberculosis* to increase its virulence, and this strategy might be modulated differently by diverse strains.

The realization of the MTBC diversity in all its extent has also led to multiple studies exploring the impact of this variation on the clinical outcome of TB (Figure 3). For example, Lineage 2 strains (which includes the Beijing family) have been repeatedly associated with treatment failure and relapse (106–108). One *in vitro* study further demonstrated that Lineage 2 may acquire drug resistance more rapidly than Lineage 4 (109), in line with reports from clinical settings where multidrug-resistant (MDR) isolates were more likely to belong to the Beijing lineage than to Lineage 4 (110, 111). High transmissibility of Beijing family Lineage 2 strains was shown in San Francisco (112), whereas Lineage 3 was reported to be less transmissible than Lineages 1, 2, and 4 in TB patients from Montreal, Canada (113), and striking differences in transmissibility among sublineages of Lineage 5 in Benin and Nigeria were reported (114). Lineages 2 and 4 were shown to have a higher rate of progression to active TB as compared with Lineage 6 in the Gambia (115). In addition, more debilitating symptoms (such as weight loss) have been associated with Lineage 4 strains in Tanzania (116). The same lineage was linked mainly to pulmonary TB (117), while Lineages 2 (117–119) and 3 (120) were reported to associate with extrapulmonary disease. Strikingly, within-host bacterial diversity also seems to contribute to disease manifestation. Indeed, the presence of distinct bacterial subpopulations is associated with poor clinical outcomes (121), resulting in differential resolution of granulomas (122), that may even contrast the overall trend of disease progression (123–125).

## Bacteria and Host Genotype Interactions

There is emerging evidence showing an impact of host–pathogen genotype interactions in host immune responses, TB transmission, and disease presentation (76). This is the case of the association observed between the presence of the T597C allele of the toll-like receptor (TLR) 2 gene and susceptibility to disseminated disease, in a Vietnamese population upon infection by MTBC Lineage 2 (117). Three studies conducted in Ghana further support genotype–genotype interactions. First, the variant G57E of the mannose-binding Lectin (Protein C) 2 (*MBL2*) gene was associated with TB caused by *M. africanum*, but not by *M. tuberculosis sensu stricto* (126). Second, the variant 261TT of the immunity-related GTPase M (*IRGM*) gene was protective against TB caused by Lineage 4, but not for disease caused by other MTBC lineages (127). Third, *M. africanum* was significantly more common in TB patients belonging to the Ewe ethnic group, an association mainly driven by Lineage 5 (128, 129). In a South African population of mixed ancestry, an association between different HLA class I types and disease caused by different MTBC strain families was reported (130). Polymorphisms in the macrophage receptor with collagenous structure (*MARCO*) gene, a receptor involved in *M. tuberculosis* phagocytosis (131), preferentially associated with Lineage 2 over Lineage 1 or 4, implying that the host MARCO genotypes may interact with *M. tuberculosis* of the Lineage 2 genotype to increase susceptibility to TB (132). The first report of a *M. tuberculosis* lineage-based GWAS was recently published (133). In this study, an SNP on chromosome 1p13, near the *CD53* gene, was specifically associated with non-Beijing lineage-infected old age onset cases (133). Altogether, these studies demonstrate that interactions between bacterial and human genetic loci exist and jointly influence clinical phenotypes. This evidence calls for the need of integrating the pathogen genotype in human genetic association studies, as well as in the study of TB immunity.

## Rewiring the Immune Response in TB: The Need to Incorporate Diversity in Host, Pathogen, and Environment

Host-directed therapies are gaining momentum in the field of TB treatment (Table 1). HDTs aim at modulating host inflammation as a way to improve the efficacy of current treatments, while shortening the duration of these treatments, lowering toxicity and decreasing rates of resistance acquisition (134). A particular benefit of HDTs would be their application to treat MDR- and extensively drug resistant-TB, where antibiotics have limited effectiveness and immunopathological inflammation, tissue damage and high fatality are observed.

**Table 1:** Evidence supporting HDTs for TB.

HDT mechanism	Examples of potential HDTs agents	Evidence on host effect	Reference
Reducing excessive tissue damaging inflammation	Ibuprofen (NSAIDs)^a^	Inhibits prostaglandin production by inhibiting cyclooxygenase. Reduces lung pathology and *Mycobacterium tuberculosis* burden in mouse models	Vilaplana et al. (135)

	Zileuton (leukotriene synthesis inhibitors)^a^	Inhibits lipoxygenase activity, blocking leukotriene production, and increasing PGE2 levels. Prevents type I IFN-driven acute mortality of *M. tuberculosis*-infected mice	Mayer-Barber et al. (136)

	Tofacitinib (tyrosine kinases inhibitors)^a^	JAK blocker with anti-inflammatory properties (JAK/STAT pathway is downstream the activation of most cytokine receptors), shortens the time required to lung sterility in a chronic TB mouse model	Maiga et al. (137)

	Adalimumab (anti-TNFα)^b^	Life-threatening pulmonary TB attributable to the recovery of TNF-dependent inflammation caused by withdrawal of adalimumab. Lung inflammation worsened despite clearance of viable *M. tuberculosis* from sputum and lung tissue by antimicrobial therapy. Clinical improvement did not occur until adalimumab treatment was resumed	Wallis et al. (138)

	Prednisolone (glucocorticoids)^d^	Modulate extreme immunopathological reactions and improved mortality for TB pericarditis and meningitis. Possible benefit in pulmonary TB. Adjunctive treatment with corticosteroids may improve the clinical outcome and may accelerate sputum smear conversion from HIV coinfected patients	Evans (139); Critchley et al. (140); Bilaçeroğlu et al. (141); Mayanja-Kizza et al. (142)

Modulating innate and adaptive immune responses	Simvastatin (statins)^a^	Inhibits the 3-hydroxy-3-methylglutaryl coenzyme reductase, reducing the cholesterol levels within phagosomal membranes, which promotes phagosomal maturation and autophagy. Reduces bacterial burden in human PBMCs and MDMs. Improves histopathologic findings, with reduced lung *M. tuberculosis* burdens in experimental murine infection	Parihar et al. (143)

	Carbamazepine (anticonvulsants)^a^	Sodium-channel blocker, capable of enhancing autophagic killing of intracellular *M. tuberculosis* in macrophages through cellular myoinositol depletion. In mice infected with a highly virulent MDR strain, carbamazepine treatment reduced bacterial burden, improved lung pathology, and stimulated adaptive immunity	Schiebler et al. (144)

	Metformin (biguanides, antidiabetic drugs)^c^	Interrupts the mitochondrial respiratory chain, increases production of mitochondrial reactive oxygen species, and facilitates phagosome–lysosome fusion, leading to enhanced killing of intracellular *M. tuberculosis*. In the mouse model, T cell responses and the efficacy of conventional TB drugs are improved, with resultant reduced lung pathology. In two separate human cohorts, metformin associates with decreased TB severity and improved clinical outcome in active TB and is associated with enhanced *M. tuberculosis*-specific T cell immune response in LTBI	Singhal et al. (145)

	Vitamin D3^d^	Induces the gene expression of beta-defensin 2 and human cathelicidin LL-37 that are able to suppress the growth of *M. tuberculosis* and modulate antimicrobial responses. Adjunct therapy with vitamin D3 enhanced intracellular mycobacterial killing in macrophages, increased sputum culture conversion, and reduced clinical symptoms in TB patients	Mily et al. (146); Rahman et al. (147)

Immune checkpoint inhibition	Nivolumab and pembrolizumab (anti-PD-1)^a^	PD-L1 gene expression is elevated in patients with active TB disease. Human gene expression of PD-1 and PD-L1 in whole-blood decrease during successful TB treatment. Infections with live *M. tuberculosis* upregulated PD-L1 expression on monocytes. *In vitro* PD-1 blocking rescued *M. tuberculosis*-specific IFN-γ-producing T cells from undergoing apoptosis. PD-1 blockade potentiates the specific degranulation of CD8+ T cells	Singh et al. (148); Jurado et al. (149); Hassan et al. (150)

Immune activation, cytokine therapy	Recombinant human IFN-γ^b^	IFN-γ administration in a patient with MSMD caused by IL-12Rβ1 deficiency provided a noticeable clinical effect, with no additional adverse effects	Alangari et al. (151)

Cell-based therapy	Autologous BM-MSCs^d^	BM-MSCs have immunomodulatory properties that can reduce damaging inflammation, induce tissue regeneration, and restore productive immune responses. Single-dose autologous BM-MSC is a safe adjunct therapy for patients with MDR or XDR-TB in combination with standard drug regimens and reconstituted anti-*M. tuberculosis* T cell responses in a phase 1 trial	Skrahin et al. (152)

Antimicrobial-potentiating effect	Verapamil (calcium-channel blockers)^a^	Blocks efflux pump, resulting in higher intracellular antimycobacterial drug levels and enhanced drug activity. Accelerates both the bactericidal and the sterilizing activities of the regimen in a mouse model. Adjunctive use of verapamil decreases the MIC of bedaquiline in the wild-type strain *M. tuberculosis* H37Rv and also in drug-susceptible and drug-resistant clinical isolates. Potentiates the activity of bedaquiline against *M. tuberculosis* in an *in vivo* mouse model. Permits lower doses of bedaquiline and thereby reduce its dose-related toxicities	Gupta et al. (153)

*BM-MSCs, bone marrow-derived mesenchymal stromal cells; MDMs, monocyte-derived macrophages; MDR, multidrug resistant; MIC, minimum inhibitory concentration; PBMCs, peripheral blood mononuclear cells; NSAIDs, non-steroidal anti-inflammatory drugs; XDR, extensively drug resistant; TB, tuberculosis; LTBI, latent TB infection; HDT, host-directed therapy; IFN, interferon; MSMD, Mendelian susceptibility to mycobacterial disease; IL, interleukin; TNF, tumor necrosis factor; PGE2, prostaglandin E2; HIV, human immunodeficiency virus*.

*Following are the types of studies*.

*^a^Preclinical*.

*^b^Case reports*.

*^c^Observational studies*.

*^d^Randomized trials*.

Although HDTs take advantage of immunomodulatory mechanisms, they rarely account for the interaction between the diversity of the bacteria, the host, and the environment. However, in some clinical sites, indirect data suggest that successful HDTs have to be tailored toward these variables. For example, there is a large amount of data showing Lineage 2 strains as being low-cytokine inducers, but potent inducers of type I IFN (97, 98, 104, 154), which is at least in part mediated by the differential activation of TLR2 and TLR4 receptors (98). In this way, Lineage 2 strains may subvert antituberculous host defenses by inhibiting the enzyme inducible NO synthase, as well as IL-1β, IL-18, and IL-12p40, while inducing the immunosuppressive mediators IL-10 and IL-1 receptor antagonist (4, 155). In addition, there are data from TB patients in Vietnam showing an association between individuals with a T597C allele in the *TLR2* gene and susceptibility to infection by the Lineage 2/Beijing genotype (117); and an association of individuals with an SNP in the leukotriene A4 hydrolase (*LTA4H*) promoter with an excess of inflammation and TB severity (156). How all the combinations of human/pathogen genotypes interact in Vietnam remains to be elucidated, but it will surely impact the outcome of HDTs in TB, as well as teaching important lessons. Furthermore, HDTs will need to deal with our partial understanding of what constitutes protective immunity to TB. For example, the role of type I IFNs is now being reconsidered. A recent study showed that in certain scenarios, namely, in the absence of IFN-γ, the induction of type I IFN is actually protective to the host, as it allows the control of the switch of M1 (effector) to M2 (detrimental) macrophages (157). The fact that type I IFN plays dynamic roles during infection (157, 158) may have implications in the use of eicosanoid modulators to enhance prostaglandin E2 levels and decrease the unfavorable type I IFN response (136). So, HDTs designed to modulate excessive type I IFN response, like the one associated with Lineage 2 strains, will need to consider host-specific characteristics, such as a high or low ability of differentiating IFN-γ responses, as well as the time of intervention.

## Conclusion

The host immune status is tightly linked to the spectrum of TB infection and disease. However, the molecular determinants bridging inflammatory thresholds and TB outcomes remain elusive. Likely, this is due to the fact that what finally dictates disease outcomes and transmission is not a single factor, but the interacting (antagonistically or synergistically) action of multiple factors. Diversity in host, pathogen, and extrinsic factors needs to be studied in concert rather than individually, so that the full extent of the biological interplay underlying the immune response can be captured and modeled. In the long term, these models will lead the discovery of solid correlates of protection, biomarkers of prognosis, therapeutic targets, and more accurate epidemiology models. The great challenge is now to integrate this *troika* of interactions in the development of HDTs, at a personalized level, to develop sterilizing therapies. Most likely, we will be soon talking about personalized medicine to treat TB, by rewiring the immune response through host–pathogen–environment directed therapies. Only then will we be able to translate the many years of research devoted to the study of the protective immune response into real clinical applications, i.e., better vaccines, therapeutics and novel biomarkers of prognosis.

## Author Contributions

All authors conceived and wrote the paper. SG, IC, and MS coordinated and completed the final version.

## Conflict of Interest Statement

The authors declare that the research was conducted in the absence of any commercial or financial relationships that could be construed as a potential conflict of interest.

## Abbreviations

CAMP, cathelicidin antimicrobial peptide; COPD, chronic obstructive pulmonary disease; DCs, dendritic cells; GWAS, genome-wide association studies; HDTs, host-directed therapies; HIV, human immunodeficiency virus; IFN, interferon; IL, interleukin; iNOS, inducible NO synthase; IRGM, immunity-related GTPase M; LTA4H, leukotriene A4 hydrolase; LTBI, latent tuberculosis infection; MARCO, macrophage receptor with collagenous structure; MBL2, mannose-binding lectin 2; MDR, multidrug resistant; MSMD, Mendelian susceptibility to mycobacterial disease; MTBC, *Mycobacterium tuberculosis* complex; NO, nitric oxide; PGE2, prostaglandin E2; SNP, single-nucleotide polymorphisms; TB, tuberculosis; Th, T helper; TLR, toll-like receptor; TNF, tumor necrosis factor; VDR, vitamin D receptor; XDR, extensively drug resistant.
